# Role of Transient Receptor Potential Ion Channels and Evoked Levels of Neuropeptides in a Formaldehyde-Induced Model of Asthma in Balb/c Mice

**DOI:** 10.1371/journal.pone.0062827

**Published:** 2013-05-09

**Authors:** Yang Wu, Huihui You, Ping Ma, Li Li, Ye Yuan, Jinquan Li, Xin Ye, Xudong Liu, Hanchao Yao, Ruchong Chen, Kefang Lai, Xu Yang

**Affiliations:** 1 Section of Environmental Biomedicine, Hubei Key Laboratory of Genetic Regulation and Integrative Biology, College of Life Sciences, Central China Normal University, Wuhan, China; 2 State Key Laboratory of Respiratory Disease, Guangzhou Institute of Respiratory Disease, Guangzhou Medical University, Guangzhou, China; 3 College of Basic Medical Sciences, Hubei University of Science and Technology, Xianning, China; University of Medicine and Dentistry of New Jersey, United States of America

## Abstract

**Objective:**

Asthma is a complex pulmonary inflammatory disease characterized by the hyper-responsiveness, remodeling and inflammation of airways. Formaldehyde is a common indoor air pollutant that can cause asthma in people experiencing long-term exposure. The irritant effect and adjuvant effect are the two possible pathways of formaldehyde promoted asthma.

**Methodology/Principal Findings:**

To explore the neural mechanisms and adjuvant effect of formaldehyde, 48 Balb/c mice in six experimental groups were exposed to (a) vehicle control; (b) ovalbumin; (c) formaldehyde (3.0 mg/m^3^); (d) ovalbumin+formaldehyde (3.0 mg/m^3^); (e) ovalbumin+formaldehyde (3.0 mg/m^3^)+HC-030031 (transient receptor potential ankyrin 1 antagonist); (f) ovalbumin+formaldehyde (3.0 mg/m^3^)+ capsazepine (transient receptor potential vanilloid 1 antagonist). Experiments were conducted after 4 weeks of combined exposure and 1-week challenge with aerosolized ovalbumin. Airway hyper-responsiveness, pulmonary tissue damage, eosinophil infiltration, and increased levels of interleukin-4, interleukin-6, interleukin-1β, immunoglobulin E, substance P and calcitonin gene-related peptide in lung tissues were found in the ovalbumin+formaldehyde (3.0 mg/m^3^) group compared with the values seen in ovalbumin -only immunized mice. Except for interleukin-1β levels, other changes in the levels of biomarker could be inhibited by HC-030031 and capsazepine.

**Conclusions/Significance:**

Formaldehyde might be a key risk factor for the rise in asthma cases. Transient receptor potential ion channels and neuropeptides have important roles in formaldehyde promoted-asthma.

## Introduction

Over recent decades, asthma has become an increasingly prevalent disease. It now represents a serious public-health problem worldwide, with an estimated 300 million people of all ages affected (especially children) [1]. Asthma is a common chronic disease of the airways characterized by enhanced airway hyper-responsiveness (AHR), reversible airway remodeling and chronic airway inflammation, which can lead to recurrent episodes of wheezing, breathlessness, chest tightness and cough [2]. Asthma is considered to be primarily an atopic disease [3]. At the cellular level, allergens are internalized by antigen-presenting cells. CD4 T_H_2 cells are then activated, resulting in the release of T_H_2-associated cytokines. This action leads to the synthesis of immunoglobulin (Ig) E antibody. This is followed by the degranulation of mast cells and infiltration of the airway mucosa with eosinophils, which induces tissue remodeling and AHR [4], [5].

Immunological inflammation has a key role in the development of asthma, but does not fully account for the complex inflammatory processes in the airways of asthmatics subjects. Authors have stated that pro-inflammatory neuropeptides are also involved in airway inflammation and AHR [6], [7]. Pro-inflammatory neuropeptides such as tachykinin substance P (SP) and calcitonin gene-related peptide (CGRP) can activate their specific receptors and induce inflammatory cells in the airway to release inflammatory mediators such as cytokines, oxygen radicals and histamine. These mediators potentiate tissue injury, stimulate the further recruitment of leukocytes, produce and amplify inflammatory responses in the airway, and participate in respiratory disease (including chronic obstructive pulmonary disease (COPD) and asthma): this is referred to as “neurogenic inflammation” [8]–[10].

The innervation of the airways is supplied by the autonomic nervous system; the autonomic nerves contribute to the regulation of airway smooth muscle tone and the transport of fluid across the bronchial epithelium [11].The largest portion of mammalian airway-innervating sensory nerve fibers originates from vagal ganglia, and a smaller number of airway sensory nerves originate from dorsal root ganglia. The cell bodies of vagal sensory fibers are located in the jugular and nodose ganglia with projections peripherally to the airways and centrally to the solitary tract nucleus in the brain stem [12].Some airway-specific neurons within the vagal sensory ganglia have relatively larger cell body diameters give rise to faster conducting myelinated A-fibers, while others with small diameter cell bodies that give rise to unmyelinated C-fibers [13]. The bronchial C-fibers are present within the airway mucosa, and the pulmonary C-fibers are located in the lung parenchyma. C-fibers can be activated by inflammatory mediators or exogenous chemical irritants, and releases various neuropeptides, in particular SP and CGRP [14]. The terminals of nerve fibers and the receptors for these neuropeptides are localized in the vessel walls, bronchial smooth muscles, the epithelial area and around mucus glands, so local stimulation of sensory neurons projecting to these targets and the subsequent neuropeptide release can lead to the features of inflammation such as hyperemia, edema, mucus hypersecretion and contraction of bronchial smooth muscle [15].

Neuropeptides have been described having a neuronal origin, but there is increasing evidence that these peptides may be synthesized and released from immune cells such as macrophages, lymphocytes and monocytes [16]–[19].Inflammatory cytokines may increase the expression of neuropeptide genes in inflammatory cells, so that inflammatory cell become a major source of the neuropeptide at the inflammatory site [20].

Transient receptor potential (TRP) channels are a group of ion channels located mostly on the plasma membrane of sensory nerve cells and other cell types. They are distributed widely within the respiratory tract. Here they act as a mechanistic link between exposure to noxious irritants and inflammation to heightened sensitivity to airway reflexes, pathological remodeling and airflow limitation, as well as being associated with allergic asthma and irritant-induced asthma [21]. TRP channels can be opened by a wide range of exogenous chemical-irritant stimuli to elicit acute pain and neurogenic inflammation through the peripheral release of neuropeptides [22], [23]. The two important pro-inflammatory TRP ion channels are TRPV1 (capsaicin receptor) and TRPA1 (activated by mustard oil and garlic) [24]–[26] because almost all major neuronal inflammatory pathways converge on TRPV1 and TRPA1 to increase the excitability of C-fibers during airway inflammation [27], and they are also present on non-neuronal cells [21], [28].

Ascertaining the risk factors for the increase in the prevalence of asthma is important. Formaldehyde (FA) is used in many products, including resins, plywood, tobacco, particle board and tissue fixatives [29], [30]. Because of its wide use and varied sources, individuals are exposed to gaseous FA in occupational and domestic environments. Acute and chronic exposure to FA has been associated with various toxic effects, including irritation, oxidative stress, nasopharyngeal cancer and respiratory disorders [31]–[33]. Several epidemiological studies have demonstrated that FA exposure can induce or exacerbate asthma [34]–[37]. Unlike typical allergens, FA-specific IgE is rarely detected [38], [39], which suggests that FA may be involved in asthma *via* a non-IgE-mediated mechanism. Two possible pathways for FA-induced asthma have been proposed: the irritant effect and the adjuvant effect [40]. Some animal studies have shown that FA alone can up regulate the levels of IgE and interleukin (IL)-4 and increase the eosinophil count [41]–[43], suggesting that FA alone may cause airway inflammation *via* an irritant effect. Animal studies have demonstrated that repeat exposure to FA can enhance the response to antigens [44]–[46], suggesting that FA acts as an adjuvant to common allergens in animal experiments. However, the mechanisms of such adjuvant effects are incompletely understood.

Recent evidence suggests that TRPV1 and TRPA1 channels can be activated by FA, which can lead to acute thermal hyperalgesia and neuropathic pain [47]–[49]. In the present study, we used a model of asthma in mice based on FA exposure. We evaluated hyper-responsiveness, inflammation and remodeling of the airways using selective receptor antagonists to investigate the role of TRP ion channels and neuropeptides in FA-promoted asthma. We also investigated the mechanisms of adjuvant effects in FA- promoted asthma.

## Materials and Methods

All experimental procedures were approved by the Office of Scientific Research Management of Huazhong Normal University (Huazhong, China) with a certification of Application for the Use of Animals dated 8 November 2011 (approval ID: CCNU-SKY-2011-008).

### Reagents and Kits

The TRPA1 antagonist HC-030031 (CAS 349085-38-7, 2-(1, 3-Dimethyl-2, 6-dioxo-1, 2, 3, 6-tetrahydro-7H-purin-7-yl)-N-(4-isopropylphenyl) acetamide) was purchased from Toronto Research Chemicals (Toronto, Canada). The TRPV1 antagonist capsazepine (CPZ, CAS 138977-28-3,N-[2-(4-Chlorophenyl)ethyl]-1,3,4,5-tetrahydro-7,8-dihydroxy-2H-2-benzazepine-2-carbothioamide) was purchased from Tocris Bioscience (Bristol, UK). Formalin solution (10%), methacholine (MCH), gelatinous Al (OH)_3_, and pentobarbital sodium were purchased from Sigma–Aldrich (St. Louis, MO, USA). Methylcellulose, ovalbumin (OVA) and bovine serum albumin (BSA) were obtained from Merck (Darmstadt, Germany). Mouse enzyme-linked immunosorbent assay (ELISA) kits for total IgE and OVA-IgE were purchased from Biolegend (San Diego, CA, USA). A mouse EILSA kit for OVA-lgG1 was purchased from Chondrex (Redmond, WA, USA). Mouse EILSA kits for IL-4, interferon (IFN) -γ and IL-1β were purchased from Raybiotech (Norcross, GA, USA). Mouse ELISA kits for SP and CGRP were purchased from R&D Systems (Minneapolis, MN, USA). Rabbit anti-SP-antibody, rabbit anti-CGRP- antibody, goat-anti-rabbit lgG-antibody, a rabbit lgG peroxidase conjugated streptavidin-biotin complex (SABC-POD) kit and a diaminobenzidine (DAB) kit were obtained from Boster Bio-engineering (Wuhan, China).

### Animals

Male Balb/c mice (5–6 weeks; 22±1.5 g) were purchased from the Hubei Province Experimental Animal Center (Wuhan, China) and housed in standard environmental conditions (12-h light–dark cycle, 50–70% humidity and 20–25°C). Food and water were provided *ad libitum*. Mice were quarantined for ≥7 days before study initiation**.** Eight mice in each group were used so as to minimize the number of experimental animals needed while ensuring the validity of statistical power.

### Preparation of Gaseous FA

Gaseous FA was prepared from 10% formalin. The solution was administered to mice through a vapor generator and automated mouth–nose only inhalation exposure chamber HOPE-MED 8052 (Hope-Med, Tianjin, China) capable of emitting gaseous FA at the concentrations demanded. Environmental parameters were monitored and controlled automatically by the system. Air temperature, relative humidity and airflow rate were maintained at 22±2°C, 50±5% and 1.65±0.15 m^3^/h, respectively. During exposure, FA concentrations were monitored every 2 h using a Gaseous FA Analyzer (4160-2, Interscan, Simi Valley, CA, USA). The level of gaseous FA adopted was 3.0 mg/m^3^ in accordance with the occupational threshold limit in China [50].

### Experimental Protocol

Mice were divided randomly into six experimental groups of 8 animals. They were exposed to or not exposed to gaseous FA for 4 weeks (days 1 to 28) at 6 h/day and 5 days/week in the inhalation exposure chamber. They were sensitized with OVA+Al(OH)_3_ (80 µg OVA and 1.3 mg gelatinous Al (OH)_3_ in 200 µL saline each time or saline (200 µL) saline each time) by subcutaneous injection on days 10, 18 and 25 followed by aerosol challenge in 1% OVA (30 min/day) or saline from days 29 to 35 using an Ultrasonic Nebulizer (402AI, Yuyue, Beijing, China). For therapeutic intervention with a TRP channel antagonist, mice were pre-administered (i.p.) with 150 µL of 8 mg/kg HC-030031 (suspended in 0.5% methylcellulose) or 1.6 mg/kg CPZ (TRPV1 dissolved in 2.5% EtOH) or vehicle 30 min before FA exposure starting each day from days 1 to 28.

**Figure 1:**
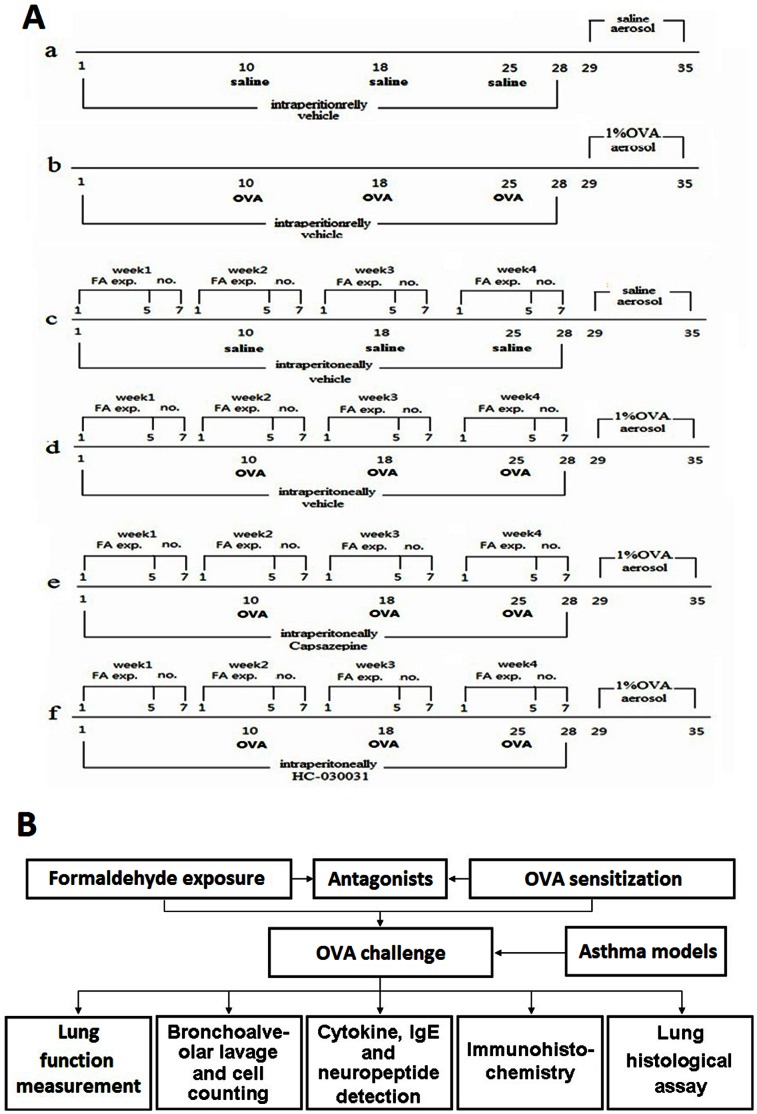
Study protocol. (A) Exposure, immunization and antagonist schedule. Groups: (a) vehicle control, (b) OVA, (c) FA, (d) FA+OVA, (e) FA+OVA+HC-030031 and (f) FA+OVA+CPZ; (B) experimental design.

Dedicated groups of mice (for each of the regimens described above) were employed to prevent inaccuracies upon measurements of lung cytokines/pulmonary architecture due to the influence of the MCH used in AHR assessments. One set of 48 mice was treated for 35 days according to the regimen(s) stated above and then used for AHR tests and collection of bronchoalveolar lavage fluid (BALF) samples. A second set of 48 mice was treated as described above and then used directly for ELISA, histopathologic and immunohistochemical analyses. The detailed protocol of this study is shown in **Fig. 1**.

### Measurement of AHR

AHR as an indicator of lung function was tested 24 h after the final exposure to aerosol using an AniRes 2005 Lung Function system (Bestlab 2.0, Beijing, China) according to manufacturer instructions. Anesthesia was induced by intraperitoneal injection of 95 mg/kg pentobarbital sodium. A connection was made by a computer-controlled ventilator *via* a cannula that had been implanted surgically in the trachea. The respiratory rate and the time ratio of expiration/inspiration were pre-set at 90/min and 1.5∶1, respectively. An injector needle was inserted into the jugular vein through which 0.025, 0.05, 0.1 and 0.2 mg/kg body weight MCH was injected successively at 5-min intervals. After each injection, airway resistance and lung compliance (Cdyn) in response to increasing concentrations of MCH were recorded by the system. Airway resistance is defined as the pressure driving respiration divided by flow. Lung compliance refers to the distensibility of the lung and is defined as the alteration in volume of the lung produced by a change in pressure across the lung [51]. Airway responsiveness was assessed by indexes of expiratory resistance (Re), inspiratory resistance (Ri), and the peak value of Cdyn. The relative area (R-area, RL) was defined as the area under the peak curve of Re or Ri and beyond the baseline level.

### Differential Cell Counts in BALF

BALF was collected immediately after the conclusion of AHR measurements. The lungs of each mouse were lavaged *in situ* with three successive 1-mL volumes of saline instilled by syringe. After gentle pressing of the chest several times, BALF was collected and combined. The recovery ratios of different mice were ≈90%. All samples were centrifuged at 100×*g* for 10 min at 4°C. The cell pellets from BALF were suspended in saline for the cell counts. Total cells, eosinophils, lymphocytes and neutrophils in cell suspensions were counted using a Blood Cell Analysis system (MTN-21, Motenu, Changchun, China).

### Quantitative Analyses of IgE in Serum

Twenty-four hours after the final challenge, blood was taken from the hearts of the mice. Then, serum samples were collected after centrifugation of the blood (3000×*g*, 15 min, 4°C) and stored at –70°C until analyses. The serum levels of total IgE (T-lg E), OVA-IgE and OVA- IgG1 were measured using an ELISA kit according to manufacturer protocols. Concentrations were determined in duplicate for each sample. The sensitivities of the ELISA kits were 0.1 ng/mL for T-lgE, 20.7 pg/mL for OVA-lgE and 0.4 ng/mL for OVA-lgG1. The coefficient of variation (CV) of ELISA kits was <8% (intra-assay) and <11% (inter-assay) for T-lgE, <4% (intra-assay) and <5% (inter-assay) for OVA-lgE, <10% (intra-assay) and<4% (inter-assay) for OVA-lgG1.

### Quantitative Analysis of Cytokines and Neuropeptides in Lung Tissue

After serum sample collection, right lungs tissue were rinsed in ice-cold phosphate-buffered saline (PBS: pH 7.5), then dried and weighed. Afterwards, they were homogenized using a glass homogenizer on ice, with 10 mL/g of ice-cold PBS. Next, homogenates was centrifuged at 2300×*g*, for 10 min at 4°C, and supernatants collected for detection using ELISA. The tissue levels of IL-4, IFN-γ, IL-1β, SP and CGRP were measured using ELISA kits according to manufacturer instructions. Concentrations were determined in duplicate for each sample. The sensitivities of ELISA kits were 0.6 pg/mL for IL-4, 5 pg/mL for IFN-γ and IL-1β, 31.5 pg/mL for SP and 3.9 pg/mL for CGRP. The CV of ELISA kits were <10% (intra-assay) and <12% (inter-assay) for IL-4, IL-1β and IFN-γ, <9% (intra-assay) and<13% (inter-assay) for SP, <8% (intra-assay) and <10% (inter-assay) for CGRP γ.

### Lung Histological Assay

Left lungs from non-lavaged mice were isolated for preparation of histopathology slices. Lung lobes were incubated in fixative (saturated 2, 4, 6-trinitrophenol: formalin: glacial acetic acid [15∶5∶1 *v/v/v*]) for 24 h at room temperature, then cut into pieces. Slides of lung tissues were stained using hematoxylin and eosin (H&E) [52], periodic acid-Schiff (PAS) [53], and Masson’s trichrome (MT) [52] methods to examine the magnitude of airway inflammation, mucus production, and peribronchial collagen deposition according to recommended standard protocols. Stained pieces were embedded in paraffin, sectioned into 10-µm slices and observed using a DM 4000B microscope (Lecia, Berlin, Germany). Tissue sections were examined qualitatively by two experienced pathologists in a blinded fashion.

### Immunohistochemical Assay

Sections of lung tissues were quenched of endogenous peroxides with 3% H_2_O_2._ They were boiled in sodium citrate (0.01 mol/L, pH 6.0) for antigen retrieval to unmask antigen epitopes, permeabilized with 0.2% Triton X-100 for 10 min, and blocked with 5% BSA in PBSfor 30 min at room temperature. Sections were incubated with diluted primary antibodies (rabbit anti-SP antibody or rabbit anti-CGRP antibody, 1∶200 dilution) overnight at 4°C. Slides were washed with PBS, incubated with secondary antibody (goat anti-rabbit IgG; 1∶200 dilutions) for 30 min at 37°C and detected with a rabbit IgG peroxidase conjugated streptavidin-biotin complex (SABC-POD) kit, followed by incubation with a diaminobenzidine (DAB) kit. Immunostained sections were viewed under a DM 4000B Microscope (Leica). The intensity of protein SP or CGRP staining was determined as average optical density using Image-Pro Plus 6.0 software (Media Cybernetics, Bethesda, MD, USA). A non-stained region was selected and set as the background. All tissue sections were examined qualitatively by two experienced pathologists in a blinded fashion.

### Statistical Analyses

All data are the mean ± standard error of the mean (except the data for substance P and calcitonin gene-related peptide measured by EILSA which are the mean ± standard deviation of the mean). Statistical graphs were generated using Origin 8.0 Software (OriginLab, Berkeley, CA, USA). One-way ANOVA combined with Fisher’s protected *t*-test was used to determine the significance of differences between groups. p<0.05 was considered significant. Data analyses were carried out using SPSS ver13 (SPSS, Chicago, IL, USA).

## Results

### Effect of TRP Channel Antagonists on AHR

**Figure 2:**
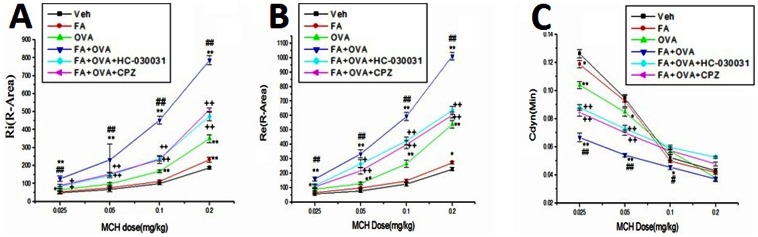
Airway hyperresponsiveness (AHR) measurements. With different doses of MCH: (A) R-area of Re. (B) R-area of Ri; (C) the peak value of Cdyn. Animal groups (in all panels): n = 3 mice per group.*: p<0.05, **: p<0.01, compared with vehicle control; #: p<0.05, ##: p<0.01, compared with OVA-immunized group; +: p<0.05, ++: p<0.01, compared with FA+OVA group.

**Figure 2** shows the results of measurements of airway responsiveness. The R-area of the respiratory resistance (RL, including Re and Ri) and peak values of Cdyn have been adopted as sensitive indices. Three parameters of lung function (Ri, Re and Cdyn) were recorded after each injection of MCH (0.025, 0.05, 0.1 and 0.2 mg/kg). In all groups, the expiratory and inspiratory resistance increased with increasing MCH levels (p<0.01), whereas Cdyn decreased (p<0.01). OVA-sensitized groups exhibited a greater airway response to MCH compared with the vehicle control group (p<0.01) and FA exposure significantly enhanced the degree of airway reactivity in OVA-sensitized mice (p<0.01). Treatment with the TRPA1 antagonist HC-030031 and TRPV1 antagonist CPZ dramatically reduced Ri, Re and restored Cdyn in FA+OVA-treated mice in response to MCH, respectively (p<0.01).

### Effect of TRP Channel Antagonists on Leukocyte Levels in BALF

**Figure 3:**
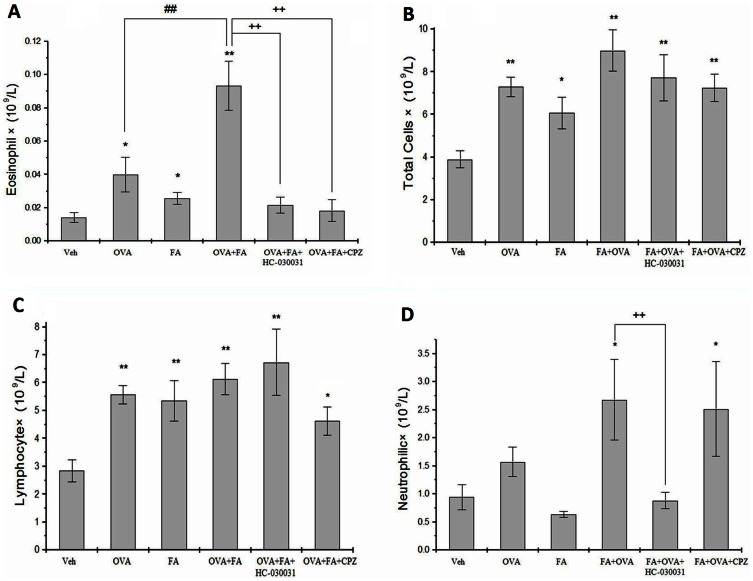
Leukocyte infiltration in the airways in different experimental groups. (A) eosinophil counts in BALF. (B) Total cell counts in BALF. (C) Lymphocyte counts in BALF. (D) Neutrophil counts in BALF. Animal groups(in all panels): veh:n = 7,OVA:n = 6,FA:n = 7,FA+OVA:n = 6,FA+OVA+HC-030031:n = 6,FA+OVA+CPZ:n = 6.*: p<0.05, **: p<0.01, compared with vehicle control; ##: p<0.01, compared with OVA-immunized group. ++: p<0.01, compared with FA+OVA group.

Levels of inflammatory cells (eosinophils, total cells, lymphocytes, neutrophils) in BALF were elevated significantly in OVA-challenged mice compared with control mice except for neutrophils (total cells: p<0.01 eosinophils: p<0.05 lymphocytes: p<0.05) (**Fig. 3** **)**. FA exposure also significantly increased the eosinophil count and total cells compared with vehicle controls (p<0.05). OVA immunization significantly intensified the increased eosinophil infiltration induced by FA (p<0.01). Compared with the control group, FA+OVA+HC-030031 and FA+OVA+CPZ treated groups have higher count of total cells (p<0.01) and lymphocytes (p<0.05), and FA+OVA+CPZ treated groups had a higher count of neutrophils (p<0.05). Treatment with CPZ and HC-030031 significantly reduced the number of eosinophils compared with the FA+OVA group (p<0.01).

### Effect of TRP Channel Antagonists on Serum Levels of IgE

**Figure 4:**
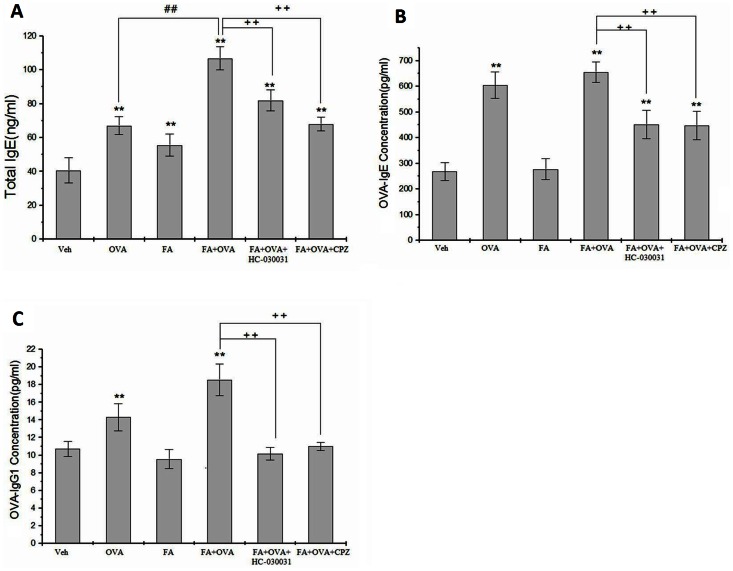
Serum IgE level. (A) Total IgE.Animal groups: veh: n = 8,OVA:n = 7,FA:n = 7,FA+OVA:n = 7,FA+OVA+HC- 030031:n = 7,FA+OVA+CPZ:n = 7.(B) OVA-IgE.Animal groups: veh: n = 7,OVA:n = 8,FA:n = 7,FA+OVA:n = 7,FA +OVA+HC-030031:n = 7,FA+OVA+CPZ:n = 7.(C) OVA-IgG1.Animal groups: veh:n = 8,OVA:n = 7,FA:n = 7,FA+. OVA:n = 7.FA+OVA+HC-030031:n = 7,FA+OVA+CPZ:n = 7.**: p<0.01, compared with vehicle control; ##: p<0.01, compared with OVA-immunized group. ++: p<0.01, compared with FA+OVA group.

**Figure 4A** shows that, compared with the vehicle control group: OVA-sensitized groups enhanced T-IgE (p<0.01); FA-only exposure promoted the level of T-IgE; and the OVA+FA group caused a significant increase in serum T-IgE (p<0.01). Treatment with CPZ and HC-030031 significantly reduced these levels compared with the FA+OVA group (p<0.01).

From the results of the analyses of OVA-IgE and OVA-IgG1 in **Figs. 4B and 4C**, respectively, exposure to FA only did not cause changes in the serum levels of OVA-IgE and OVA-IgG1 (p>0.05). OVA-only groups demonstrated significantly of increased levels OVA-IgE and OVA-IgG1 in relation to vehicle controls (p<0.01). Levels of OVA-IgE and OVA-IgG1 were significantly inhibited by the administration of TRP channel antagonists (p<0.01).

### Effect of TRP Channel Antagonists on Cytokines Levels

**Figure 5:**
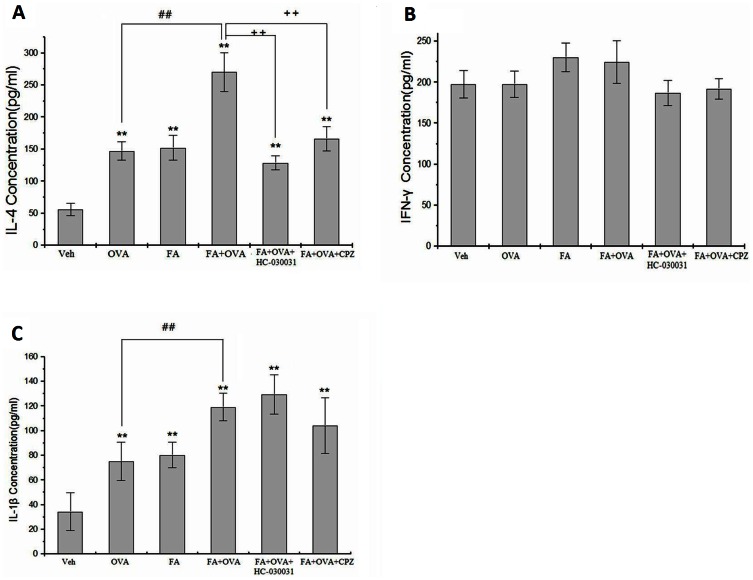
Cytokine levels in lung tissue. (A) IL-4 levels. Animal groups: veh: n = 6,OVA:n = 7,FA:n = 8, FA+OVA:n = 7, FA+OVA+HC-030031:n = 6,FA+OVA+CPZ:n = 6. (B) IFN-γ levels. Animal groups: veh: n = 8,OVA:n = 8,FA:n = 8; FA+OVA:n = 7, FA+OVA+HC-030031:n = 6,FA+OVA+CPZ:n = 6. (C) IL-1β levels. Animal groups:veh: n = 7,OVA: n = 7;FA:n = 8;FA+OVA:n = 8,FA+OVA+HC-030031:n = 6,FA+OVA+CPZ:n = 6. **: p<0.01, compared with vehicle control; ##: p<0.01, compared with OVA-immunized group. ++: p<0.01, compared with FA+OVA group.

The levels of the Th2 cytokine IL-4 and the Th1 cytokine IFN-γ were assessed in samples of lung tissue. The FA exposure group and OVA-sensitized group induced a significant increase in IL-4 levels (p<0.01). Among all groups, exposure to FA+OVA group induced the highest IL-4 levels (p<0.01). Treatment with CPZ and HC-030031 significantly reduced IL-4 levels compared with FA+OVA groups (p<0.01) (**Fig. 5A**). However, IFN-γ levels were not significantly different among all groups (**Fig. 5B**).

Exposure to FA and OVA enhanced the levels of the pro-inflammatory cytokine IL-1β in lung tissue (p<0.01) (**Fig. 5C**). Exposure to the FA+OVA group induced the highest IL-4 levels (p<0.01). Treatment with CPZ or HC-030031 did not reduce these levels compared with FA+OVA groups (p>0.05).

### Effect of TRP Channel Antagonists on Neuropeptide Release in the Airways

**Figure 6:**
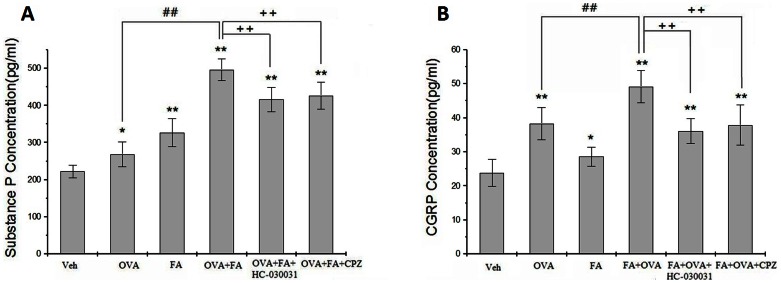
Neuropeptide levels in lung tissue. (A) Substance P. Animal groups: veh: n = 6,OVA:n = 6, FA+OVA:n = 6, FA+OVA:n = 6, FA+OVA+HC-030031:n = 6,FA+OVA+CPZ:n = 6. (B) CGRP. Animal groups: veh: n = 7,OVA:n = 8, FA+OVA:n = 7, FA+OVA:n = 8, FA+OVA+HC-030031:n = 7,FA+OVA+CPZ:n = 7.*: p<0.05, **: p<0.01, compared with vehicle control; ##: p<0.01, compared with OVA-immunized group. ++: p<0.01, compared with FA+OVA group.

FA exposure and OVA-sensitized groups enhanced the levels of SP and CGRP in the lungs (p<0.05) (**Fig. 6** **)**. Exposure to FA+OVA induced the highest levels of release (p<0.01). Treatment with CPZ and HC-030031 significantly reduced the levels of SP (HC-030031 group, F = 20.67, p<0.01; CPZ group, F = 13.66, p<0.01) or CGRP (HC-030031 group, F = 35.45, p<0.01; CPZ group, F = 17.11, p<0.01) compared with FA+OVA groups.

**Figure 7:**
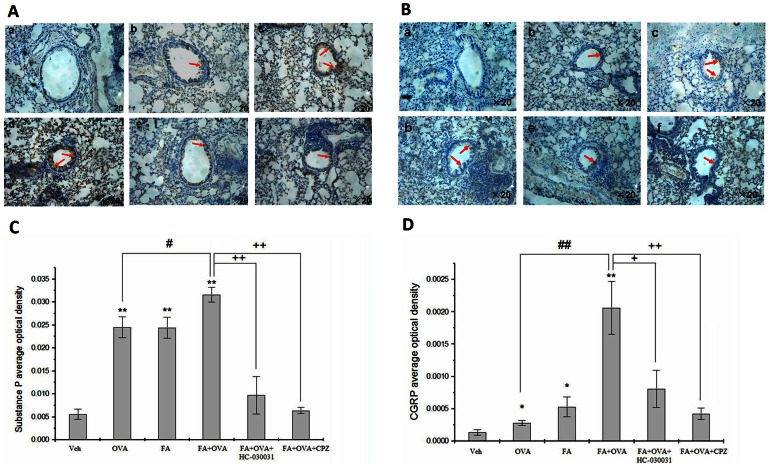
Immunohistochemical analyses. Representative images of the expression of (A) Substance P and (B) CGRP as determined by immunohistochemical staining (brown color stain) (red arrow). Panel: (a) negative control group, (b) OVA group, (c) FA group, (d) FA+OVA group, (e) FA+OVA+HC-030031 group, and (f) FA+OVA+CPZ group; Magnification = ×20. Analyses of (C) Substance P and (D) CGRP expression levels according to average optical density. Animal groups (in all panels): n = 4 mice per group.*: p<0.05, **: p<0.01, compared with vehicle control; #: p<0.05, ##: p<0.01, compared with OVA-immunized group. +: p<0.05, ++: p<0.01, compared with FA+OVA group.

Immunohistochemical analyses were conducted to detect the expression of SP and CGRP in lung tissue. SP and CGRP were expressed mainly in the epithelial layers around the bronchioles (**Figs. 7A and 7B**). Expression of these immunoreactive proteins was up-regulated in the OVA-treated group and FA-treated group compared with the control groups. Exposure to FA+OVA induced the highest levels. However, when mice were treated with HC-030031 or CPZ, expression of SP and CGRP in lung tissues was restored.

The average optical density was also measured. We noted in **Figs. 7C and 7D** that the OVA group presented an increase in the content of SP and CGRP, respectively, compared with the control group(p<0.01; p<0.05); and FA exposure significantly enhanced the level of SP and CGRP in lung tissue(p<0.01). FA exposure significantly enhanced the levels of SP and CGRP in OVA-sensitized mice (p<0.05; p<0.01), HC-030031 and CPZ reduced such content when compared with the OVA group (p<0.05).

### Effect of TRP Channel Antagonists on Airway Remodeling

**Figure 8:**
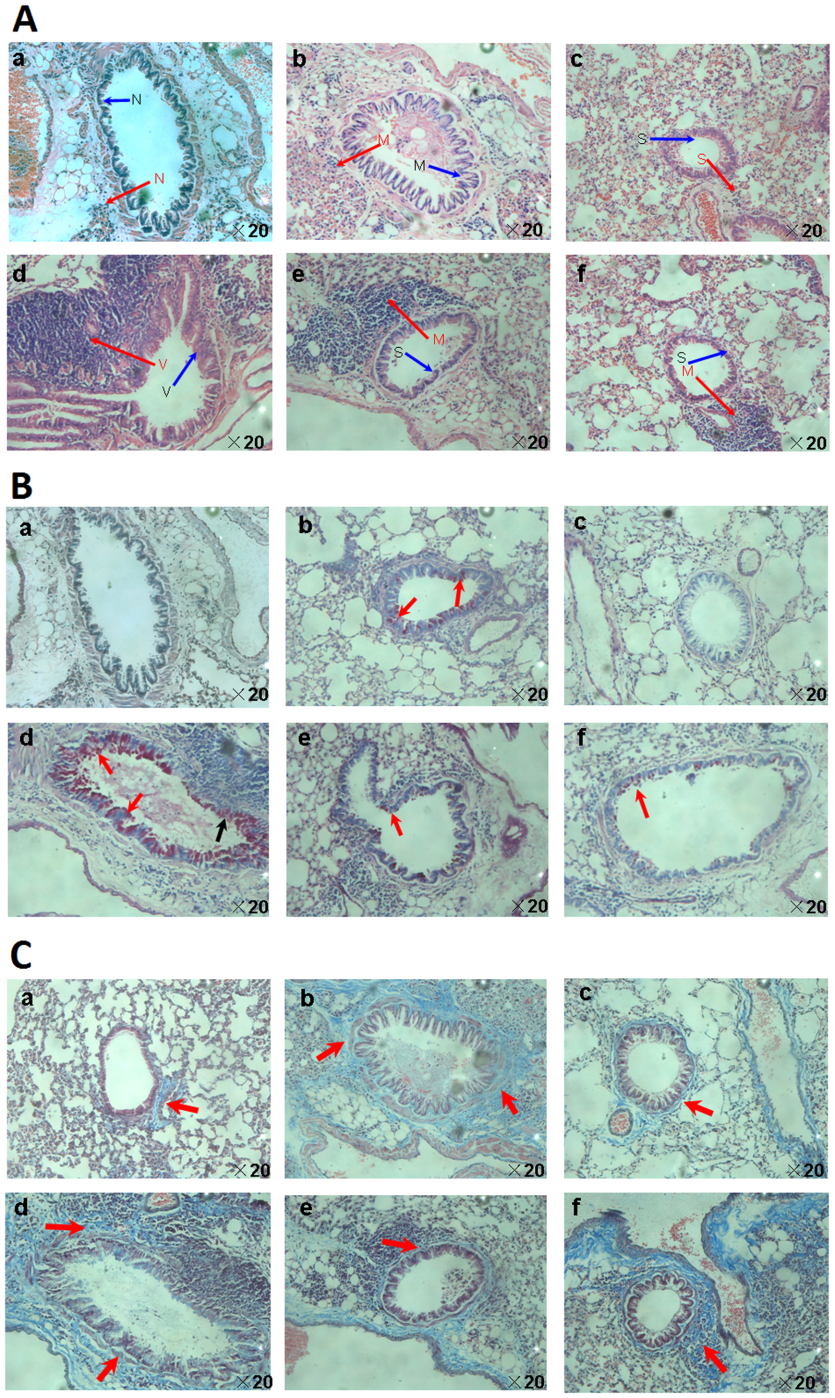
Representative histological images of airway remodeling of lung tissue. (A) H&E staining: showing infiltration of inflammatory cells (red arrow) and hypertrophy of structural cells (blue arrow). N: normal conditions; S: slight changes; M: moderate changes; V: severe changes. (B) Periodic acid-Schiff (PAS) staining: showing mucous cells (pale pink color stain) (red arrow). (C) Masson’s trichrome (MT) staining showing subepithelial collagen deposition (blue color stain) (red arrow). Panel: (a) vehicle control group, (b) OVA group, (c) FA group, (d) FA+OVA group, (e) FA+OVA+HC-030031 group and (f) FA+OVA+CPZ group. Magnification = ×20.Animal groups (in all panels): n = 4 mice per group.

The increase in airway responsiveness and inflammation in mice was associated with histopathological changes in the bronchiolar airways. The typical pathological features of airway inflammation and structural alterations in asthmatic were observed: infiltration of inflammatory cells in the peribronchial and perivascular areas (H&E staining) (**Fig. 8A**). Mucus overproduction and hyperplasia of goblet cells (PAS staining) (**Fig. 8B**) and peribronchial deposition of collagen (MT staining) (**Fig. 8C**) were observed in OVA-challenged mice. Slight changes were observed in the FA group compared with the vehicle control group. The most severe changes in airway structure were in the FA+OVA group. Treatment with CPZ and HC-030031 markedly reduced the degree of infiltration of inflammatory cells, mucus overproduction, goblet-cell hyperplasia, peribronchial deposition of collagen and fibrosis.

## Discussion

Here we reported that the TRPA1 channel antagonist HC-030031 and TRPV1 channel antagonist CPZ reduced FA exposure in allergen-induced inflammation, pulmonary tissue damage and AHR. We also revealed that SP and CGRP participate in the development of FA-promoted asthma or asthma-like symptoms.

The expression of asthma is an intricate process dependent upon the interaction between two major factors: environmental exposure and genetic factors [54]. The present study showed that pre-administration of low levels of HC-030031 and CPZ during FA exposure and an OVA-sensitized phase was sufficient to potently suppress: AHR; leukocyte infiltration; production of Th2 cytokines and IgE; neuropeptide release; pulmonary physiological changes. These findings suggest that TRPA1 and TRPV1 have an important role in FA-promoted asthma.

To investigate the effects of FA exposure on the structure and function of bronchial airways, we used a murine model of asthma based on a well-established OVA sensitization/challenge protocol [43], [46]. In the present study, to simulate environmental exposure in the workplace, exposure for 5 days/week and 6 h/day was adopted, and 4 weeks of a low dose of FA was administered rather than immediately high-dose exposures before allergen challenge. For smaller rodent species (e.g., mouse), using nose-only modes in inhalation studies is considered superior to whole-body exposure. This is because the nose-only route can reduce dosing from non-respiratory routes (including wall loss as well as losses on the skin and fur of animals), which cause the actual concentration to be substantially less than the nominal concentration. Most applications of selective receptor antagonists are by direct injections, particularly peritoneal injection, which is convenient to carry out. The injection dose was decided according to the results in our preliminary experiments before the formal ones.

Hyper-responsiveness and remodeling of the airways are the major pathophysiological features of asthma, which lead to airway narrowing and airflow obstruction. The degree to which AHR can be defined by contractile responses to challenges with MCH correlates with asthma severity. MCH as a pharmacological agent can cause increased Ri/Re and decreased Cdyn. The R-areas of respiratory resistances (Ri and Re) explain the variation in the large airways, whereas the change in Cdyn represents the state of the small airways or the parenchyma [51]. Airway remodeling is difficult to define because it involves several structural changes. These include thickening of the sub-basement membrane, hyper-secretion of mucus, subepithelial fibrosis, infiltration of inflammatory cells, and deposition of the extracellular matrix in the subepithelial layer. In the present study, we used three types of staining methods to reveal changes in pulmonary histology. H&E staining is a classical and typical staining research method for airway remodeling. It can be used to examine the magnitude of airway inflammation, but staining of collagen and goblet cells of the lung sections cannot be done based on H&E staining. Hence, we used PAS methods to examine mucus secretion and goblet cells, and using MT methods to examine peribronchial collagen deposition to describe airway remodeling.

The FA exposure group showed increased AHR compared with the control group and caused slight, but statistically significant, histological changes in the airways of mice. However, these changes were not obviously different compared with those seen in the OVA group. However, upon co-exposure with OVA, AHR and airway remodeling was exacerbated.

The main pathological feature of asthma is allergic airway inflammation, and an imbalance of Th1/Th2 immune responses is a pathological basis of asthma and other atopic diseases [55]. IFN-γ represents the Th1 immune response and is associated with protective immunity; IL-4 represents the Th2 immune response, drives the type-2 pathway and upregulates IgE production to fight extracellular organisms. In the present study, IFN-γ levels were not significantly different among the experimental groups. Levels of IL-4 and T-lgE were markedly increased in OVA-immunized mice exposed to FA. We observed up-regulated expression of IgE and IL-4 in FA-alone groups; this finding may reflect acceleration of the imbalance of Th1/Th2 cytokines and contribute to the cause and evolution of asthma.

Increased numbers of eosinophils in peripheral blood and in airway secretions are a characteristic feature of asthma [56] and correlate with a greater severity of asthma. Eosinophils then pass from the circulation *via* the microvascular compartment into the airway wall. Data from the present study showed that the number of eosinophils was markedly increased in OVA-immunized mice exposed to FA. Hence, the eosinophil count is a major biomarker for FA-promoted asthma.

Pro-inflammatory cytokines activate the immune system and participate in the inflammatory response. IL-1β is an important pro-inflammatory cytokine released by innate immune cells during inflammation. IL-1β is increased in the bronchial epithelium and macrophages of patients with asthma [57], and IL-1β is required for OVA-specific IgE production and induction of eosinophilic airway inflammation [58]. The present study showed that the co-existence of allergen (OVA) and FA can increase the highest level of IL-1β. Hence, IL-1β may have a key role in FA-promoted asthma. Our data also showed IL-1β levels were not inhibited by HC-030031 or CPZ, thereby suggesting that IL-1β expression is not mediated by TRP channels.

We confirmed our previous findings that an adjuvant effect contributes to the development of FA-induced asthma or asthma-like symptoms [43], [46]. Additionally, a significant difference in the levels of OVA-IgE and OVA-IgG1 was found between the OVA+FA and OVA-alone group.

The results of ELISA and immunohistochemical analyses suggested that the OVA-treated group modulated the release of SP and CGRP; these results are consistent with those in other animal studies [59], [60].These data may suggest that allergen exposure stimulates immune cells to produce neuropeptides. Neuropeptides can activate the immune system, influence the activity of immune cells and stimulates: degranulation of mast cells and release histamine and 5-HT; T lymphocyte and macrophages to release pro-inflammatory cytokine, macrophages and eosinophils to produce and secrete neuropeptides [61]. Thus, our data in sensitized animals suggest that immune interactions could be mediated by neuropeptides. Immunity is coordinated by neural circuits that operate reflexively, exogenous molecular products interact with receptors that are expressed by cells of the innate immune system, activate innate immune responses and induce the secretion of pro-inflammatory cytokines(e.g. IL-1β).This in turn activates the efferent arc, which is known as the “cholinergic anti-inflammatory pathway” [62]. Release of SP and CGRP led to vasodilation and plasma extravasation as well as bronchoconstriction and mucus production, respectively. Hence, neuropeptide release and neurogenic inflammation may have a role in the development of asthmatic symptoms and amplify the immune response during allergic inflammation [63].

The present study showed that the level of SP and CGRP was markedly increased in OVA-immunized mice exposed to FA, and that this pathway could be blocked by TRPA1 and TRPV1 channel antagonists, These findings suggested that FA inhalation enhances neuropeptide levels in a TRP ion channel-dependent way. TRPV1 is expressed not only in neurons but also in mast cells [64], [65], but further more studies needed to identify the cell origin.Evidence shows that TRPA1 channels identified in non-neuron cells including lymphocytes [66], and genetic deletion of TRPA1 drastically attenuates inflammation and hyper-reactivity in a mouse model of asthma [67]. TRP channels are involved in different immune functions, which are relevant to asthma pathophysiology, thereby, suggesting a role for TRP proteins.

In conclusion, the present study suggested that FA enhances the sensitization of Balb/c mice to inhaled allergens and that it might be an underlying risk factor for an increase in asthma severity; Further, TRPV1, TRPA1 and neuropeptides play a key part in the adjuvant effect of FA-promoted asthma or asthma-like symptoms.
